# Equivalent Clinical Outcomes but Divergent Biological Adaptation After Anterior Cervical Discectomy and Fusion Versus Cervical Disc Arthroplasty: A Longitudinal MRI Cohort Study

**DOI:** 10.3390/jcm15156116

**Published:** 2026-08-06

**Authors:** Evren Sönmez, Lokman Ayhan, Said Onar, Ergin Anlı, Abdurrahim Tekin, Engin Can, Selçuk Yapar, Akın Öztürk, Suna Dilbaz, Nuri Serdar Baş, Serdar Çevik

**Affiliations:** Department of Neurosurgery, İstanbul Kanuni Sultan Süleyman Training and Research Hospital, University of Health Sciences, 34303 İstanbul, Türkiye; op.dr.lokmanayhan@gmail.com (L.A.); onarmsaidmd@gmail.com (S.O.); anliergin@hotmail.com (E.A.); canengin@outlook.com (E.C.); selcukypr@hotmail.com (S.Y.); akozturk@gmail.com (A.Ö.); sunadilbaz@gmail.com (S.D.); nuriserdarbas@gmail.com (N.S.B.); dr.serdarcevik@gmail.com (S.Ç.)

**Keywords:** anterior cervical discectomy and fusion, cervical disc arthroplasty, cervical degenerative disc disease, magnetic resonance imaging, paraspinal muscles, fatty infiltration

## Abstract

**Background/Objectives:** Anterior cervical discectomy and fusion (ACDF) and cervical disc arthroplasty (CDA) can produce comparable short-term clinical outcomes, but their regional effects on cervical muscle morphology are incompletely characterized. This study compared 12-month clinical outcomes and quantitative magnetic resonance imaging (MRI) changes after single-level C6–7 ACDF and CDA. **Methods:** This retrospective longitudinal cohort included 100 age-, sex-, and body mass index-matched patients (50 ACDF and 50 CDA). Neck and arm pain were assessed on 0–10 scales, and the Neck Disability Index (NDI) was analyzed as a 0–50-point score. Regional muscle cross-sectional area (CSA) and fatty infiltration (FI) were quantified on standardized preoperative and 12-month axial T2-weighted turbo spin-echo images at C6–7. The primary analysis included four posterior muscles. CSA change was expressed relative to baseline, whereas FI change was expressed in percentage points. **Results:** Clinical outcomes were statistically indistinguishable at 12 months (neck-pain score, 0.50 vs. 0.36, *p* = 0.318; NDI, 2.6 vs. 2.2 points, *p* = 0.479; perfect-outcome rate, 68.0% in both groups). Both groups showed significant regional posterior muscle CSA reduction and FI increase; however, the magnitude was greater after ACDF than after CDA (CSA reduction, −15% to −19% vs. −5% to −9%; absolute FI increase, +5.1 to +8.2 vs. +1.1 to +2.3 percentage points; all between-group q < 0.001). Surgical technique was independently associated with both MRI change metrics. Greater regional CSA reduction was modestly associated with higher concurrent 12-month neck-pain scores (β = −0.26, *p* = 0.022). This concurrent association does not establish causation or predict outcomes beyond 12 months. **Conclusions:** ACDF and CDA produced equivalent measured clinical outcomes at 12 months, while CDA showed a more favorable regional posterior muscle MRI profile. Differential device-related artifact was not quantitatively assessed and cannot be completely excluded, although concordant findings across four implant-remote posterior muscles make artifact unlikely to be the sole explanation. The functional and longer-term clinical significance of this imaging difference is not established and requires prospective longitudinal study.

## 1. Introduction

Cervical degenerative disc disease (CDDD) is among the most prevalent causes of cervical radiculopathy and myelopathy worldwide, contributing substantially to pain, disability, and healthcare utilization [1]. When conservative management fails to provide adequate relief, surgical decompression is the established standard of care [2]. Anterior cervical discectomy and fusion (ACDF) using an interbody cage has remained the reference procedure for single-level anterior cervical decompression for more than six decades and continues to demonstrate consistent, reproducible clinical outcomes [3].

ACDF, however, abolishes segmental motion at the treated level, a biomechanical consequence associated with altered kinematics and increased mechanical stress at adjacent segments; the long-term risk of adjacent segment disease (ASD) following fusion has been documented in seminal studies [4]. Cervical disc arthroplasty (CDA) was developed as a motion-preserving alternative designed to maintain physiological segmental kinematics while achieving neural decompression equivalent to that of fusion [5]. Randomized controlled trials with extended follow-up have confirmed that CDA is non-inferior to ACDF for short-term pain and disability outcomes and may offer long-term advantages with respect to ASD incidence and reoperation rates [6,7]. However, cervical arthroplasty devices vary in material composition, bearing design, constraint, motion behavior, and propensity for heterotopic ossification; findings obtained with one prosthesis should not be assumed to apply identically to all CDA designs [5,8].

This short-term clinical equivalence is defined largely by patient-reported outcomes, which may not capture differences in the regional musculoskeletal response to surgery. The cervical paraspinal musculature contributes to segmental load sharing, fine motor control, and dynamic cervical alignment [9,10]. A decrease in regional muscle CSA indicates less muscle bulk on the measured axial slice, whereas an increase in FI indicates a greater proportion of fat signal within the muscle region of interest. These directions are generally regarded as less favorable structural features in paraspinal-muscle research [11,12,13,14,15], but they are imaging surrogates and do not independently establish impaired activation, reduced strength, histological replacement, or future clinical harm.

Quantitative MRI permits reproducible measurement of regional CSA and FI after cervical spine surgery. Previous studies have largely evaluated ACDF without a motion-preserving comparator, used cross-sectional designs, or relied on qualitative assessment [9,11,12,13,14,15]. A longitudinal comparison of ACDF and CDA using matched cohorts and standardized preoperative and postoperative MRI may therefore identify procedure-associated differences that are not apparent on 12-month clinical scores. Because a single standardized axial slice was analyzed in the present study, all MRI findings are interpreted as regional morphology at the C6–7 measurement level rather than total muscle volume or global degeneration of the multisegmental cervical musculature.

The purpose of this retrospective longitudinal cohort study was to compare 12-month clinical outcomes and quantitative MRI changes following single-level C6–7 ACDF and CDA in age-, sex-, and BMI-matched patients. Secondary objectives were to identify variables independently associated with regional CSA and FI change and to explore sex-stratified patterns. We hypothesized that, despite comparable short-term clinical improvement, CDA would be associated with a smaller magnitude of regional cervical muscle CSA reduction and FI increase than ACDF.

## 2. Materials and Methods

### 2.1. Study Design and Ethical Approval

This retrospective longitudinal cohort study was approved by the Clinical Research Ethics Committee of İstanbul Kanuni Sultan Süleyman Training and Research Hospital, University of Health Sciences, İstanbul, Türkiye (Approval No. KAEK/2025.08.214). The requirement for individual informed consent was waived given the retrospective design and fully anonymized data. All procedures adhered to the ethical standards of the 1964 Declaration of Helsinki and its subsequent amendments.

### 2.2. Patient Selection

Patients who underwent single-level anterior cervical discectomy at C6–7 between January 2016 and December 2023 at a single tertiary spine center were screened. Restriction to C6–7 minimized anatomical and biomechanical heterogeneity and enabled level-specific comparison. Cervical disc arthroplasty is not offered above a defined age threshold at our institution, so the inclusion age range (25–50 years) structurally limits the population in which age most strongly drives treatment selection, reducing the age-related indication bias that propensity-score matching would otherwise need to address statistically. This design feature reduces, but does not eliminate, treatment-selection bias within the included age range.

Inclusion criteria were age 25–50 years; BMI 20–30 kg/m^2^; single-level C6–7 surgery with either a bladed PEEK interbody cage (ACDF) or the mobile-core prosthesis described below (CDA); complete 12-month clinical follow-up; and preoperative and 12-month postoperative cervical MRI adequate for paired quantitative analysis. A 12-month postoperative MRI was routinely scheduled as part of the departmental follow-up pathway irrespective of symptom status. MRI scheduling was not triggered by persistent pain, suspected complications, reoperation, failure to reach PASS, or an unfavorable VAS or NDI result. Exclusion criteria included multilevel surgery, prior cervical surgery, inflammatory arthropathy, neuromuscular or systemic musculoskeletal disease, cervical deformity, instability or trauma, and imaging that precluded reliable muscle delineation. The absence of an analyzable paired examination could reflect non-attendance or non-completion of scheduled follow-up, postoperative MRI obtained externally with a non-harmonized protocol, or inadequate image quality, including motion degradation, insufficient spatial resolution, or incomplete visualization of required muscle boundaries. Clinical outcomes and the direction or magnitude of MRI-derived muscle change were not used as eligibility criteria.

**Figure 1:**
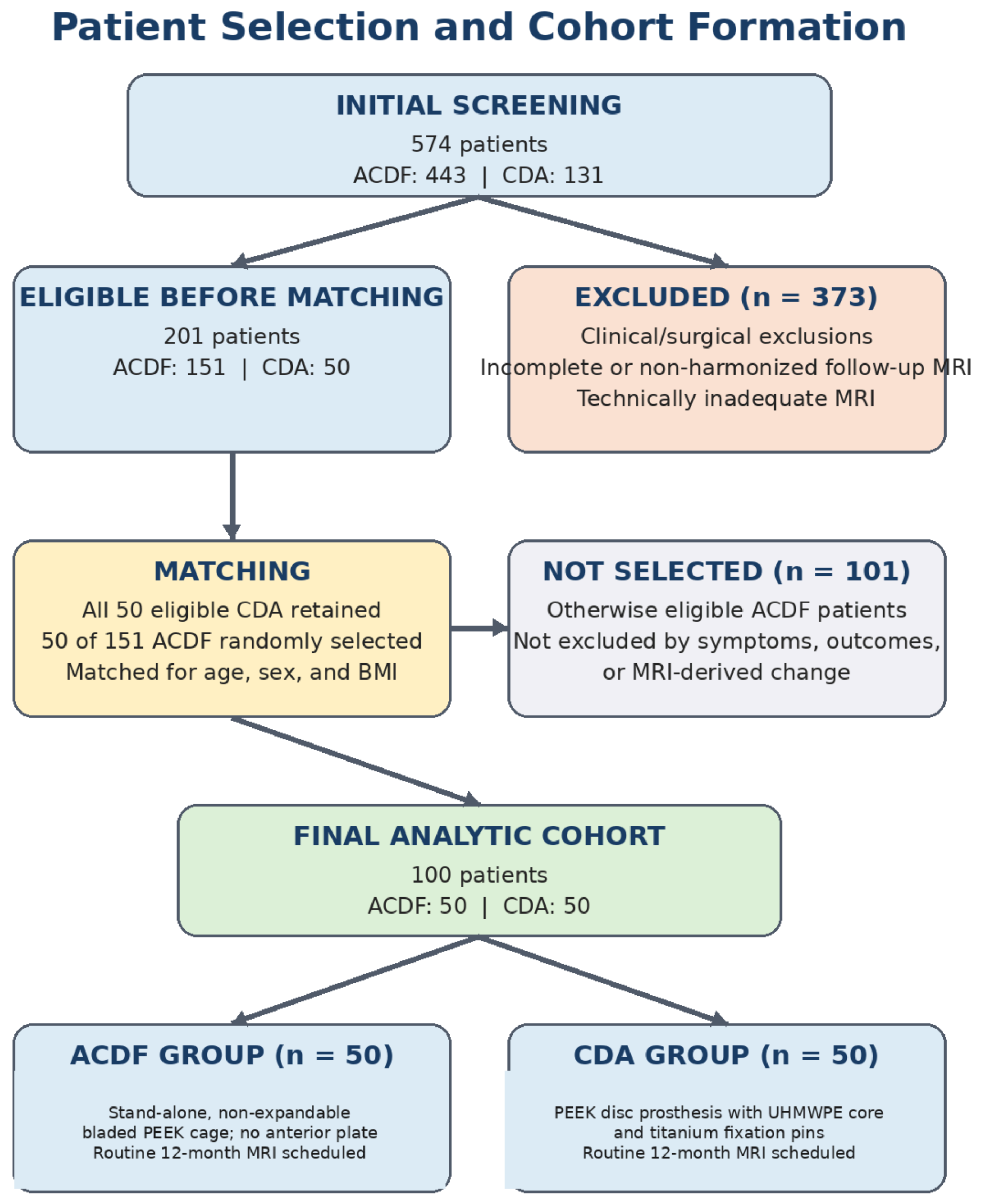
Patient selection and cohort formation. Of 574 screened patients, 201 were eligible before matching (151 ACDF and 50 CDA), indicating 373 exclusions before matching. Fifty ACDF patients were randomly selected to match all 50 eligible CDA patients for age, sex, and BMI; 101 otherwise eligible ACDF patients were not selected during matching. Routine 12-month MRI scheduling was symptom-independent. Reasons for absence of an analyzable paired MRI included incomplete follow-up, external non-harmonized imaging, and technically inadequate images; mutually exclusive counts for these categories were not reliably preserved in the historical screening record. Final inclusion still required completion of follow-up and a technically analyzable paired MRI.

A total of 574 patients were screened (443 ACDF and 131 CDA). After predefined exclusions, 201 patients were eligible before matching (151 ACDF and 50 CDA), corresponding to 373 exclusions before matching. All 50 eligible CDA patients were retained. Fifty ACDF patients were selected from the 151 eligible ACDF patients by random-number assignment in SPSS while matching age, sex, and BMI; the remaining 101 eligible ACDF patients were not selected during matching and were not imaging- or outcome-based exclusions. Age, sex, and BMI were selected a priori because they were consistently available and directly relevant to muscle morphology and recovery. A broader propensity-score model was not used because symptom duration, frailty, bone mineral density, facet degeneration, and several other potential treatment-selection variables were not recorded with sufficient completeness and standardization across the screened source population. A propensity model based on selectively available covariates would have required additional complete-case restriction, could have reduced the limited eligible CDA pool, and could not resolve unmeasured confounding. The final cohort comprised 100 patients (50 per group) (Figure 1).

### 2.3. Surgical Technique

All procedures were performed at a single institution by a limited group of experienced spine surgeons using a right-sided anterior approach and standardized institutional protocols. In the ACDF group, standard endplate preparation was followed by insertion of a stand-alone, non-expandable bladed PEEK interbody cage (Tria Spine, Ankara, Türkiye), without anterior plate fixation. In the CDA group, a mobile-core cervical PEEK disc prosthesis with a UHMWPE core and titanium fixation pins (LorX® Cervical PEEK Disc Prosthesis, Tria Spine, Ankara, Türkiye) was implanted. Both constructs were predominantly PEEK-based and incorporated only limited metallic fixation elements; however, device-specific artifact burden was not quantitatively compared in this cohort [16,17,18].

### 2.4. Postoperative Rehabilitation

Postoperative management was identical across groups. All patients wore a soft cervical collar for 4 weeks, followed by standardized home-based cervical range-of-motion exercises for up to 3 months. No formal supervised physiotherapy was prescribed; rehabilitation exposure was therefore considered equivalent between groups.

### 2.5. MRI Acquisition and Quantitative Muscle Analysis

**Figure 2:**
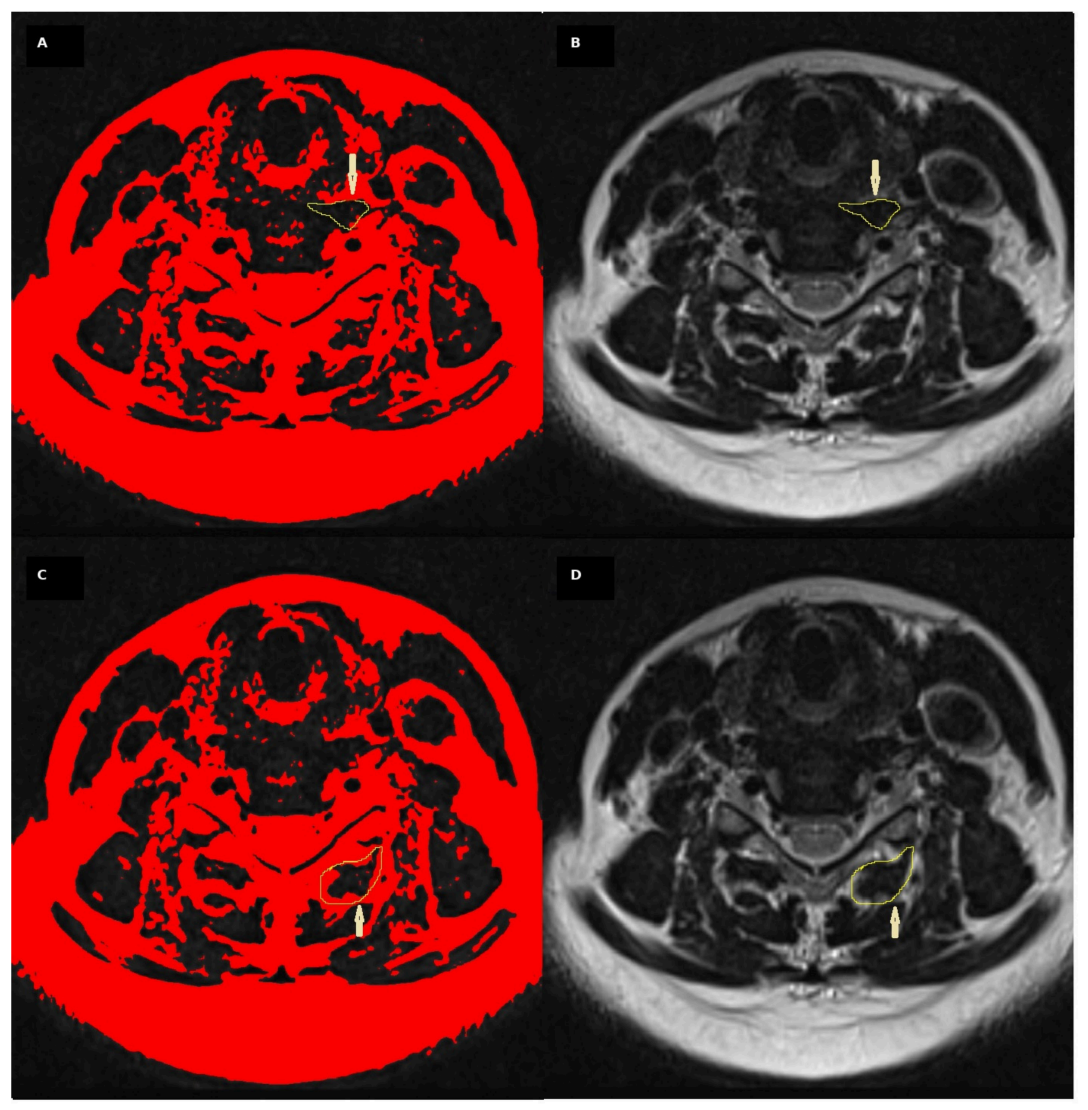
Representative axial T2-weighted MR images at the C6–7 level illustrating the muscle analysis methodology. (**A**,**B**) Longus colli (LC); (**C**,**D**) multifidus (MF). Panels (**A**,**C**) show threshold-based visualization of fatty infiltration (FI), whereas panels (**B**,**D**) show manual region-of-interest (ROI) delineation. Arrows indicate the target muscles, and yellow contours denote the corresponding ROIs. All measurements were performed using Fiji (ImageJ).

Quantitative MRI was used to characterize regional cervical muscle morphology. All examinations were performed using a 1.5 T Philips Ingenia MRI system (Philips Medical Systems, Best, The Netherlands) with standardized cervical imaging protocols. Measurements were obtained from axial T2-weighted turbo spin-echo images at the C6–7 intervertebral disc level on preoperative and 12-month studies, using the same anatomical landmark at both time points. Six muscles were evaluated bilaterally: multifidus, semispinalis cervicis, semispinalis capitis, splenius capitis, longus colli, and sternocleidomastoid (SCM). CSA (cm^2^) and FI (percentage of the muscle ROI occupied by fat signal) were quantified using Fiji (ImageJ version 1.54g; National Institutes of Health, Bethesda, MD, USA). FI was calculated using a fixed threshold-based workflow referenced to adjacent subcutaneous adipose tissue on the same axial slice [13]. CSA ROIs were delineated according to predefined anatomical boundaries; bilateral values were averaged. Because implant morphology was visible on the measurement images, observers could not be blinded to surgical technique. To limit other sources of bias, standardized slice selection and quantitative measurement were performed by different observers, and clinical outcome data (VAS, NDI, PASS, and reoperation status) were stored separately and were unavailable during image analysis. The workflow is illustrated in Figure 2. T2-weighted turbo spin-echo acquisition at 1.5 T reduces susceptibility sensitivity compared with gradient-echo imaging and higher field strength, but does not eliminate implant-related artifact [19,20,21].

The four primary muscles—multifidus, semispinalis cervicis, semispinalis capitis, and splenius capitis—are located in the posterior paraspinal compartment and are anatomically remote from the anterior implant position. Longus colli is adjacent to the operative corridor and implant, and SCM is a superficial anterior-lateral muscle; both were therefore prespecified as secondary outcomes. This anatomical hierarchy, the absence of a bulk-metal anterior plate in the ACDF group, and the low-susceptibility CDA material design reduce the likelihood that direct implant-proximity distortion alone explains concordant findings across the four posterior muscles. Nevertheless, implant artifact was not quantitatively mapped, and differential measurement bias cannot be completely excluded, particularly for the implant-adjacent longus colli. Because measurements were obtained from a single standardized axial slice, CSA and FI characterize regional morphology at the index measurement level and should not be interpreted as total muscle volume or global multisegmental degeneration.

### 2.6. Reliability Analysis

Intra- and interobserver reliability was assessed in 20 randomly selected patients. Intraobserver reliability was determined by repeat measurement after a minimum 3-week interval. ICCs with 95% CIs were calculated using a two-way random-effects model with absolute agreement [22]. The reliability subsample included both surgical groups, but estimates were pooled rather than stratified by technique. Accordingly, the ICC analysis assesses overall repeatability of the measurement protocol; it does not exclude systematic technique-related observer bias or establish equivalent device-specific measurement precision.

### 2.7. Clinical Outcome Measures

Clinical outcomes were assessed preoperatively and at 12 months using 0–10 pain scales for neck and arm pain (0 = no pain; 10 = worst imaginable pain) [23] and the NDI [24]. The NDI consists of 10 items scored from 0 to 5, yielding a raw total of 0–50 points; the source dataset stored and analyzed these raw point totals without converting them to percentages. A composite binary outcome (“perfect outcome”) was defined as neck-pain score < 1 and NDI < 5 points. A sensitivity analysis used PASS criteria of pain score ≤ 2 and NDI ≤ 15 points.

### 2.8. Statistical Analysis

Statistical analyses were performed using IBM SPSS Statistics version 25.0 (IBM Corp., Armonk, NY, USA) and Python version 3.13 (Python Software Foundation, Wilmington, DE, USA), with SciPy version 1.18.0 and statsmodels version 0.14.6. Normality was assessed with the Shapiro–Wilk test. Given non-normal muscle-change distributions, between-group comparisons used Mann–Whitney U tests and within-group changes used Wilcoxon signed-rank tests. CSA relative change was calculated for each patient as 100 × (postoperative CSA−preoperative CSA)/preoperative CSA. FI change was calculated as postoperative FI minus preoperative FI and is reported in percentage points (pp). Between-group inferential tests for FI, associated q values, and Cohen’s d estimates were based on this absolute percentage-point change. For CSA, inferential testing used absolute change in cm^2^, while relative percentages are shown descriptively. Multiple comparisons across six muscles and two parameters were controlled using the Benjamini–Hochberg FDR procedure [25]. All tests were two-sided with α = 0.05.

Multivariable linear regression assessed variables independently associated with composite posterior Δ CSA and Δ FI (primary models) and with 12-month neck-pain and NDI scores (secondary models). Each model included five prespecified predictors—surgical technique, sex, age, BMI, and the corresponding preoperative muscle composite—providing 20 observations per predictor. Standardized (β) and unstandardized (B) coefficients with 95% CIs are reported. Surgical technique was coded CDA = 1 and ACDF = 0; sex was coded male = 1. Sex-stratified comparisons within each surgical group were designated exploratory a priori and used Mann–Whitney U tests with within-group FDR correction.

## 3. Results

### 3.1. Baseline Comparability

The final cohort consisted of 100 patients (50 ACDF and 50 CDA). The groups were matched for age, sex, and BMI and were also statistically comparable for cervical lordosis, preoperative VAS and NDI scores, and preoperative CSA and FI in every evaluated muscle (all *p* > 0.05). This observed balance supports comparability for measured baseline variables but does not exclude imbalance in variables that were unavailable or not standardized in the retrospective records. Baseline characteristics are summarized in Table 1.

### 3.2. Clinical Outcomes at 12 Months

Both groups achieved favorable and statistically indistinguishable clinical outcomes at 12 months (Table 2). Mean neck-pain scores were 0.50 ± 0.8 after ACDF and 0.36 ± 0.6 after CDA (*p* = 0.318), and mean arm-pain scores were 0.12 ± 0.4 and 0.04 ± 0.2, respectively (*p* = 0.213). Mean NDI scores were 2.6 ± 3.1 and 2.2 ± 2.4 points, respectively (*p* = 0.479). Perfect-outcome rates were identical (34/50, 68.0%; *p* = 1.00), and PASS criteria were met by 98% of patients in each group.

### 3.3. Reliability of MRI-Based Muscle Measurements

Interobserver reliability was excellent for CSA (ICC = 0.88; 95% CI, 0.81–0.93) and substantial for FI (ICC = 0.82; 95% CI, 0.74–0.89). Intraobserver reliability was excellent for CSA (Observer 1: ICC = 0.93; Observer 2: ICC = 0.91) and good-to-excellent for FI (Observer 1: ICC = 0.88; Observer 2: ICC = 0.86). These pooled estimates support reproducibility of the standardized workflow across the reliability subsample but do not formally exclude a technique-specific artifact or expectation effect.

### 3.4. Within-Group Regional Muscle Change

Both groups demonstrated statistically significant regional postoperative changes from baseline across the evaluated muscles (all Wilcoxon *p* < 0.001). Across the four primary posterior muscles, ACDF was associated with mean CSA reductions of 15–19% and absolute FI increases of 5.1–8.2 pp. The corresponding changes after CDA were smaller but still statistically significant: CSA reductions of 5–9% and FI increases of 1.1–2.3 pp. Thus, regional muscle change occurred after both procedures; the procedures differed in magnitude rather than in the presence or absence of change.

### 3.5. Between-Group Differences in Regional Muscle Change

Table 3 summarizes the primary posterior muscle comparisons using one unambiguous scale for each measure: relative percentage change for CSA and absolute percentage-point change for FI. ACDF was associated with greater regional CSA reduction and FI increase across all four posterior muscles, with medium-to-large or large effect sizes that remained significant after BH-FDR correction (all q < 0.001). For multifidus, mean CSA change was −17.9% after ACDF versus −9.1% after CDA (d = 0.83), while FI increased by +8.15 versus +2.30 pp (d = 1.36). The largest absolute FI difference was observed in the multifidus, and the largest FI effect size was observed in the splenius capitis (d = 1.80). Effect sizes across primary and secondary outcomes are illustrated in Figure S1.

**Figure 3:**
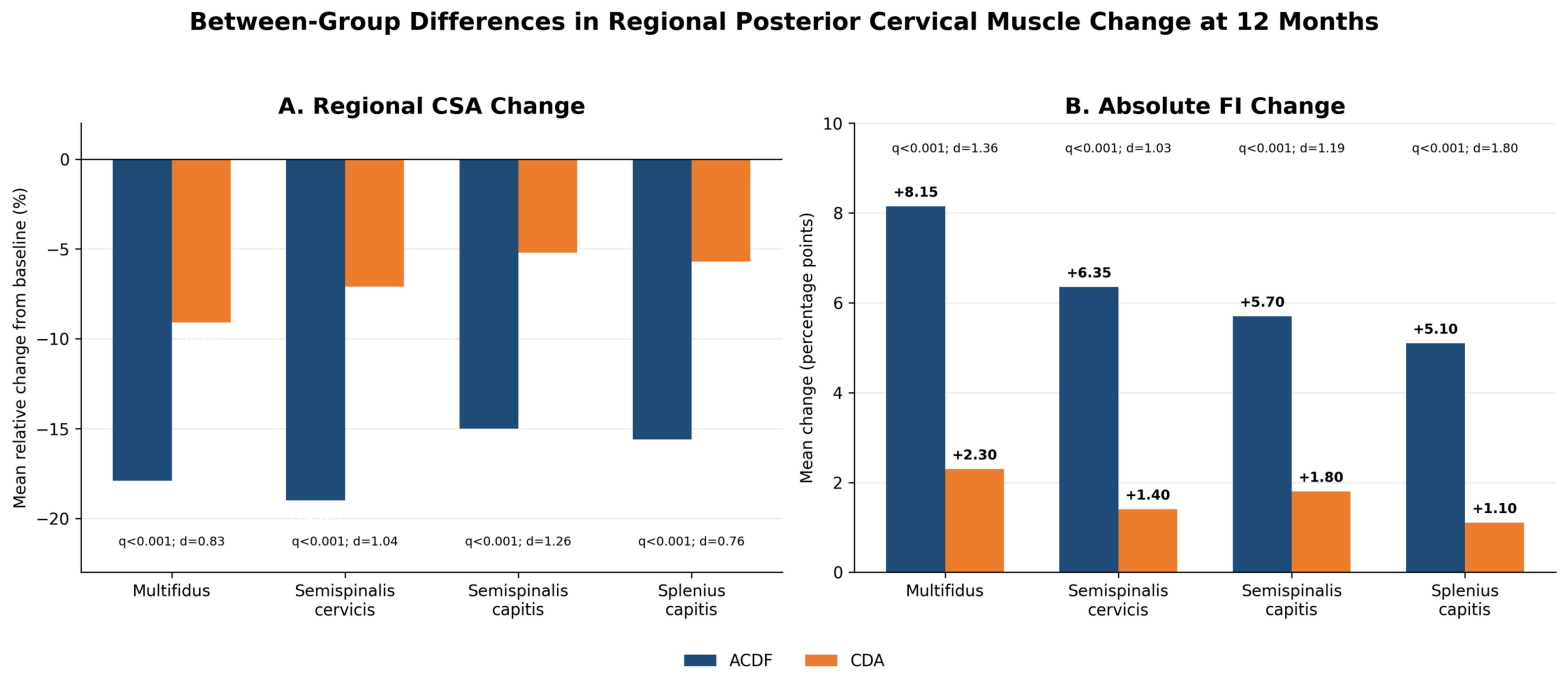
Between-group differences in regional posterior cervical muscle change at 12 months. (Panel (**A**)) mean relative change in cross-sectional area from baseline (% Δ CSA). (Panel (**B**)) mean absolute change in fatty infiltration (Δ FI, percentage points). All displayed comparisons used Mann–Whitney U tests with BH-FDR correction; q < 0.001 for every primary posterior muscle comparison. Cohen’s d values are shown for the corresponding tested metric. CSA = cross-sectional area; FI = fatty infiltration.

Among secondary outcomes, longus colli CSA reduction was greater after ACDF than after CDA (−18.8% vs. −6.3%; q < 0.001; d = 1.10), and the absolute FI increase was also greater (+3.87 vs. +0.84 pp; q < 0.001; d = 1.41). Because longus colli is adjacent to the operative corridor and implant, this finding is secondary and more susceptible to retraction- or artifact-related bias. SCM CSA reduction was small but greater after ACDF (−2.8% vs. −1.7%; q < 0.001; d = 0.66), whereas SCM FI change did not differ (+0.27 vs. +0.24 pp; q = 0.153; d = 0.19). Figure 3 displays the four primary posterior muscle comparisons using relative Δ CSA and absolute Δ FI in percentage points.

### 3.6. Factors Associated with Postoperative Muscle Change and 12-Month Clinical Outcomes

Surgical technique was independently associated with both composite posterior Δ CSA (β = 0.57, B = +10.84 percentage points of relative CSA change, *p* < 0.001) and Δ FI (β = −0.81, B = −4.82 pp, *p* < 0.001). With CDA coded 1 and ACDF coded 0, B = −4.82 pp indicates an adjusted 4.82-percentage-point smaller composite posterior FI increase after CDA. Male sex was associated with a smaller CSA reduction (β = 0.19, *p* = 0.027) and a smaller FI increase (β = −0.17, *p* = 0.004). Older age (β = −0.14, *p* = 0.029) and higher preoperative FI (β = 0.16, *p* = 0.007) were also associated with Δ FI, whereas BMI was not significant in either primary model. The Δ FI model explained 67% of variance (adjusted R^2^ = 0.673). Absolute postoperative CSA was not independently associated with clinical outcomes (all *p* > 0.45). More negative Δ CSA—indicating greater regional CSA reduction—was modestly associated with higher concurrent 12-month neck-pain scores (β = −0.26, *p* = 0.022). This contemporaneous association is not a prospective prediction of future pain. Results are summarized in Table 4.

The contemporaneous association between postoperative Δ CSA and the 12-month neck-pain score is illustrated in Figure S2.

### 3.7. Sex-Stratified Subgroup Analysis

Within the ACDF group, female patients had greater CSA reduction in semispinalis cervicis (−24.2% vs. −13.8%; *p* = 0.0009; q = 0.004) and semispinalis capitis (−19.7% vs. −10.3%; *p* = 0.0003; q = 0.003) than male patients. Multifidus FI increase was also greater in women on the unadjusted comparison (+10.0 vs. +6.3 pp; *p* = 0.0209), but this comparison did not remain significant after within-group FDR correction (q = 0.056). Within the CDA group, no sex-related comparison remained significant after correction (all q ≥ 0.222). These exploratory results are summarized in Table S1 and illustrated in Figure S3.

## 4. Discussion

### 4.1. Principal Findings

The principal result is endpoint-specific. ACDF and CDA produced statistically indistinguishable pain, disability, perfect-outcome, and PASS results at 12 months; therefore, neither procedure was clinically superior within the follow-up interval studied. Both procedures were associated with significant regional posterior muscle CSA reduction and FI increase, but these changes were consistently greater after ACDF. CDA consequently showed a more favorable regional MRI muscle-morphology profile, without evidence that this imaging difference currently translates into superior patient outcomes or should determine procedure selection.

### 4.2. Clinical Interpretation of Equivalent 12-Month Outcomes

The favorable clinical results in both groups are compatible with effective neural decompression by either procedure. The concentration of 12-month scores near the favorable end of the scales may also have limited clinical discrimination. However, equivalent VAS and NDI results do not validate or invalidate the regional MRI findings, because clinical scores and imaging measures address different endpoints. The present study demonstrates concurrent clinical equivalence and an MRI morphology difference at 12 months; it does not establish that later clinical divergence will occur [14,26].

### 4.3. Graded Regional Muscle Change: Fusion Versus Motion Preservation

The significant changes observed after CDA are incompatible with a binary model in which fusion causes muscle change and arthroplasty completely prevents it. Both procedures involved anterior discectomy, operative exposure, temporary collar use, perioperative activity modification, and the same institutional rehabilitation pathway; these shared factors may contribute to the smaller but significant changes after CDA. Moreover, CDA preserves substantial index-level motion but does not recreate an unoperated native segment [27]. The treated disc is removed, a prosthesis is implanted, and regional mechanics and motor recruitment may change after either operation.

The consistently larger ACDF-associated changes across all four posterior muscles nevertheless suggest an incremental procedure-associated effect beyond the factors shared by both groups. Cervical multifidus has a specialized segmental architecture [10], and experimental or immobilization literature demonstrates that regional muscles can respond selectively and rapidly to altered loading or disuse [28,29,30]. These data support, but do not prove, a hypothesis that eliminating index-level motion may alter regional recruitment, fascicular loading, length–tension behavior, or load sharing. Muscle activation, afferent signaling, segmental kinematics, and histological composition were not measured; the mechanism therefore remains hypothesis-generating.

The anatomical pattern provides internal context but not definitive mechanistic proof. The four primary posterior muscles are remote from the anterior implants and showed concordant between-group differences. SCM FI did not differ, whereas the implant-adjacent longus colli followed the general direction of the posterior findings but was treated as secondary because anterior retraction and differential artifact are more plausible at that location. The findings are therefore best described as a graded procedure-associated difference in regional morphology, not as global atrophy or a demonstrated fusion-specific biological cascade.

### 4.4. Predictors of Adaptation and Their Relationship to Clinical Outcomes

Surgical technique showed the largest adjusted association with composite Δ CSA and Δ FI, and the group differences were not explained by the measured age, sex, BMI, or baseline muscle variables. This does not mean that technique was proven to be causal or that all confounding was removed. Treatment allocation was non-random, and unmeasured factors—including symptom duration, frailty, bone quality, facet degeneration, physical activity, and comorbidity burden—may have influenced both procedure selection and postoperative morphology.

Greater regional CSA reduction was modestly associated with higher neck-pain scores measured at the same 12-month follow-up. Because the imaging change accumulated from baseline to 12 months and pain was assessed at 12 months, this result is contemporaneous rather than prognostic. It does not establish causation, mediation, progressive deterioration, or future harm, and it should not be interpreted as evidence that the MRI difference will later become clinically apparent. A systematic review of lumbar spine surgery similarly found that postoperative muscle change may be more informative than baseline morphometry, although extrapolation across spinal regions and procedures is limited [31].

### 4.5. Sex as a Modifier of Postoperative Adaptation

Within the ACDF group, female patients showed greater CSA reduction in the semispinalis cervicis and semispinalis capitis and greater multifidus FI increase than male patients; no comparison within CDA remained significant after FDR correction. Female sex was also independently associated with greater composite FI increase. Sex-related differences in skeletal muscle response have biological plausibility [32,33], but the present subgroup analysis included only 25 patients per sex within each procedure and was not powered for a formal interaction test. The finding is exploratory and should not be interpreted as a demonstrated sex-specific response mechanism.

### 4.6. Reconciling the Magnitude of Regional Change with Prior Long-Term and Device-Specific Evidence

The magnitude of the present early regional findings requires comparison with the important long-term study by Matsumoto et al. [34]. That study followed 31 patients for a mean of approximately 12 years after one- or two-level non-instrumented anterior decompression and fusion and compared them with 32 healthy controls. Multifidus, semispinalis cervicis, semispinalis capitis, and splenius capitis were measured at C3–4, C4–5, and C5–6, and the principal analysis emphasized aggregate CSA summed across muscles and levels; C6–7 was not measured. By contrast, the present study assessed each muscle separately using paired preoperative and 12-month postoperative measurements on a single standardized axial slice at the operated C6–7 level. A focal index-level signal could therefore be diluted by aggregation across several muscles and levels, although this difference in measurement sensitivity does not prove that the long-term study missed a true effect. Matsumoto et al. also used different MRI systems and sequences at baseline and follow-up, which may have reduced sensitivity to longitudinal change but does not invalidate their result. Their absence of marked long-term posterior extensor CSA loss is counterevidence to any assumption that the 15–19% regional reductions observed here represent a universal or inevitable ACDF trajectory. The restricted age and BMI ranges in the present cohort may have reduced baseline heterogeneity and improved paired-measurement precision, but cannot by themselves explain the biological magnitude. The discrepancy therefore remains unresolved, and the present estimates should be regarded as cohort-specific 12-month C6–7 single-slice findings requiring independent replication and serial volumetric imaging. The asymptomatic longitudinal data of Okada et al. [26] further emphasize that age and measurement level influence cervical extensor morphology.

Protocol-specified postoperative MRI independent of symptom status has precedent in cervical arthroplasty research. In the multicenter FDA investigational device exemption trial reported by Guyer et al., the investigational PEEK-on-ceramic arthroplasty cohort underwent protocol-defined postoperative MRI assessment at 24 months, including evaluation of adjacent structures [16]. This provides a contextual precedent for scheduled postoperative imaging but is not presented as evidence of our institutional pathway and does not eliminate follow-up completion or image availability bias in the present retrospective cohort.

The CDA evidence is also device-specific. Contemporary reviews describe substantial variation among prostheses in material composition, bearing design, constraint, and kinematic behavior [5]. Karadağ et al. reported medium-term radiological and clinical follow-up of the Alpha-D prosthesis and showed that motion preservation and heterotopic ossification must be interpreted at the device level [8]. The present findings, therefore, apply to the PEEK mobile-core prosthesis with a UHMWPE core and titanium fixation pins used in this cohort and should not be extrapolated without qualification to materially or mechanically different devices.

### 4.7. Clinical Interpretation and Implications

The ACDF–CDA comparison should be interpreted according to the endpoint considered. Neither procedure was clinically superior at 12 months, whereas CDA showed a more favorable regional posterior muscle MRI morphology profile. The clinical consequence of this imaging difference is unproven, and it should therefore be treated as a hypothesis-generating comparative signal, not as a current basis for declaring one procedure globally superior or for changing procedure selection, prognostic counseling, or rehabilitation practice. Established considerations—including indication, facet status, adjacent-segment risk, implant longevity, heterotopic ossification, and cost—remain distinct from the uncertain clinical relevance of the present MRI findings.

### 4.8. Limitations

This study has several limitations. Its retrospective, non-randomized design precludes causal inference. Matching was limited to age, sex, and BMI, although measured baseline clinical, alignment, and muscle variables were also comparable. Residual confounding by unavailable variables such as symptom duration, frailty, bone quality, facet degeneration, physical activity, and comorbidity cannot be excluded; a broader propensity analysis was not feasible because these covariates were not recorded consistently across the source cohort, and propensity methods cannot balance unmeasured variables. The institutional age threshold and restricted study age range reduce age-related indication bias but do not eliminate treatment-selection bias within the eligible population. Routine 12-month MRI scheduling was symptom-independent, which reduces indication-driven imaging bias, but inclusion still required follow-up completion and technically analyzable paired imaging. Residual selection related to non-attendance, external non-harmonized imaging, or image quality remains possible, and the 98% PASS rate applies to the analyzed cohort rather than all screened patients.

A single axial slice at C6–7 characterizes regional morphology rather than whole-muscle volume. Observers could not be blinded to surgical technique because implant morphology was visible on the measurement images. Outcome blinding, separation of slice selection from quantitative measurement, predefined anatomical boundaries, fixed threshold-based FI quantification, bilateral averaging, and high pooled ICCs reduced opportunities for subjective variation but did not eliminate technique-related expectation bias. The reliability subsample was not designed or powered for treatment-stratified ICC estimation; therefore, pooled reproducibility cannot establish identical measurement precision between implants. Future prospective work should incorporate randomized image order, implant-masked presentation where technically feasible, automated or semiautomated segmentation, and blinded central adjudication.

The ACDF and CDA constructs differed in material composition, and device-specific artifact was not quantitatively mapped. No prospective phantom calibration, dedicated SEMAC/MAVRIC acquisition, or procedure-stratified reliability analysis was performed. A residual differential artifact effect on CSA or FI measurement cannot therefore be formally excluded, particularly for the implant-adjacent longus colli, which was treated as a secondary outcome. The principal analysis was based on four posterior muscles anatomically remote from the implants and measured at 1.5 T using T2-weighted turbo spin-echo imaging. The concordant posterior pattern and absence of a significant SCM FI difference make direct implant-proximity artifact an unlikely sole explanation, but do not prove equivalent measurement precision between devices.

The study cannot determine which shared perioperative factors contributed to the significant changes observed in both groups or isolate the incremental effect of eliminating index-level motion. Muscle activation, afferent signaling, segmental kinematics, strength, histological composition, and rehabilitation adherence were not measured. The analysis was limited to single-level C6–7 surgery, a right-sided approach, a single institutional rehabilitation pathway, and 12-month follow-up; the sex subgroup analysis was exploratory. Because the imaging and pain outcomes were assessed at the same 12-month time point, their association is contemporaneous rather than prognostic. The persistence, reversibility, progression, and longer-term clinical consequences of the MRI differences remain unknown.

## 5. Conclusions

ACDF and CDA achieved equivalent measured clinical recovery at 12 months, while both procedures showed statistically significant regional posterior muscle CSA reduction and FI increase at the C6–7 measurement level. The magnitude of these changes was consistently greater after ACDF, giving CDA a more favorable regional MRI morphology profile in this cohort. These findings support a graded procedure-associated difference rather than a categorical mechanism in which fusion alone produces change and arthroplasty prevents it.

The findings do not establish overall clinical superiority, global muscle degeneration, or an expected future trajectory. Differential device-related artifact was not quantitatively assessed and cannot be completely excluded; the implant-adjacent longus colli result is therefore secondary, although the concordant pattern across four implant-remote posterior muscles makes artifact unlikely to be the sole explanation. Because CDA devices differ in materials, mechanics, and heterotopic-ossification behavior, the comparison is specific to the prosthesis used and should not be generalized automatically to all arthroplasty designs. Prospective studies using repeated volumetric imaging, device-aware artifact assessment, and longer clinical follow-up are required to determine reproducibility, mechanism, and clinical relevance.

## Figures and Tables

**Table 1:** Baseline demographic and clinical characteristics of the ACDF and CDA groups.

Characteristic	ACDF (*n* = 50)	CDA (*n* = 50)	*p* Value
Age, years (mean ± SD)	39.9 ± 6.4	39.5 ± 6.3	0.78
Sex, male/female	25/25	25/25	1.00
BMI, kg/m^2^ (mean ± SD)	26.0 ± 3.3	25.5 ± 2.4	0.44
Cervical lordosis, ° (mean ± SD)	11.3 ± 2.9	11.4 ± 2.6	0.84
Preoperative muscle CSA/FI (all muscles) ^a^	No significant between-group difference		>0.05

SD = standard deviation; BMI = body mass index; CSA = cross-sectional area; FI = fatty infiltration. ^a^ Preoperative MRI showed no significant between-group difference in CSA or FI for any evaluated muscle (all *p* > 0.05).

**Table 2:** Clinical outcomes at 12 months, ACDF vs. CDA.

Outcome (12 Months)	ACDF (*n* = 50)	CDA (*n* = 50)	*p* Value
VAS-neck (0–10), mean ± SD	0.50 ± 0.8	0.36 ± 0.6	0.318
VAS-arm (0–10), mean ± SD	0.12 ± 0.4	0.04 ± 0.2	0.213
NDI, points (0–50), mean ± SD	2.6 ± 3.1	2.2 ± 2.4	0.479
Perfect outcome, *n* (%) ^d^	34 (68.0)	34 (68.0)	1.00
PASS criteria met, % ^e^	98	98	1.00

VAS = Visual Analog Scale; NDI = Neck Disability Index; PASS = Patient Acceptable Symptom State. Neck and arm pain were scored from 0 to 10. NDI values are raw points on the 0–50 scale, not percentages. ^d^ Perfect outcome was defined as neck-pain score < 1 and NDI < 5 points. ^e^ PASS criteria were pain score ≤ 2 and NDI ≤ 15 points; attainment was identical in both groups (98% vs. 98%; *p* = 1.00).

**Table 3:** Between-group differences in postoperative regional muscle change at 12 months, ACDF vs. CDA.

Muscle	Δ CSA ACDF (%)	Δ CSA CDA (%)	Δ FI ACDF (pp)	Δ FI CDA (pp)	q Value	Cohen’s d
Multifidus	−17.9	−9.1	+8.15	+2.30	<0.001/<0.001	0.83/1.36
Semispinalis cervicis	−19.0	−7.1	+6.35	+1.40	<0.001/<0.001	1.04/1.03
Semispinalis capitis	−15.0	−5.2	+5.70	+1.80	<0.001/<0.001	1.26/1.19
Splenius capitis	−15.6	−5.7	+5.10	+1.10	<0.001/<0.001	0.76/1.80
Longus colli (secondary)	−18.8	−6.3	+3.87	+0.84	<0.001/<0.001	1.10/1.41
Sternocleidomastoid (secondary)	−2.8	−1.7	+0.27	+0.24	<0.001/0.153	0.66/0.19

CSA = cross-sectional area; FI = fatty infiltration; pp = percentage points. Δ CSA values are mean within-group relative percentage changes from baseline. Δ FI values are mean within-group absolute changes in FI expressed in percentage points (postoperative FI minus preoperative FI). Between-group tests used absolute change values (CSA in cm^2^; FI in percentage points) with BH-FDR correction across the prespecified muscle-by-parameter comparisons. The q values and Cohen’s d estimates are reported as CSA/FI and correspond to the metrics actually tested. Negative Δ CSA denotes regional CSA reduction, and positive Δ FI denotes increased fat signal within the measured ROI; these directions are less favorable morphologically but do not independently establish functional impairment or clinical superiority.

**Table 4:** Multivariable linear regression: variables independently associated with postoperative Δ CSA and Δ FI in the composite posterior muscle group.

Predictor	β (Δ CSA Model)	*p* (Δ CSA)	β (Δ FI Model)	*p* (Δ FI)
Surgical technique	0.57	<0.001	−0.81	<0.001
Male sex	0.19	0.027	−0.17	0.004
Age	0.02	0.80	−0.14	0.029
Preoperative FI	0.01	0.92	0.16	0.007
BMI	−0.04	0.66	0.03	0.70
Model adjusted R^2^	0.322	-	0.673	-

β = standardized regression coefficient. Separate linear regression models were fitted for composite posterior Δ CSA and Δ FI, each including surgical technique, sex, age, the corresponding preoperative muscle measure, and BMI (100 patients; five predictors; 20 observations per predictor). Surgical technique was coded CDA = 1 and ACDF = 0; sex was coded male = 1. The coefficients represent adjusted associations and do not establish causation. BMI = body mass index; CSA = cross-sectional area; FI = fatty infiltration; pp = percentage points.

## Data Availability

The datasets generated and/or analyzed during the current study are not publicly available due to institutional data protection regulations, but are available from the corresponding author on reasonable request.

## Supplementary Materials

The following supporting information can be downloaded at: https://www.mdpi.com/article/10.3390/jcm15156116/s1, Figure S1: Effect sizes (Cohen’s d) for between-group differences in postoperative muscle cross-sectional area and fatty infiltration, ACDF vs. CDA at 12 months; Figure S2: Association between composite posterior Δ CSA and 1-year VAS-neck score in all patients and by surgical group; Figure S3: Sex-stratified postoperative changes in posterior muscle CSA and FI within the ACDF and CDA groups; Table S1: Sex-stratified subgroup analysis of postoperative regional muscle change within each surgical group (exploratory; *n* = 25 per sex per group).

## Abbreviations

The following abbreviations are used in this manuscript:

Abbreviation	Definition
ACDF	Anterior Cervical Discectomy and Fusion
ASD	Adjacent Segment Disease
BH-FDR	Benjamini–Hochberg False Discovery Rate
BMI	Body Mass Index
CDA	Cervical Disc Arthroplasty
CDDD	Cervical Degenerative Disc Disease
CI	Confidence Interval
CSA	Cross-Sectional Area
FI	Fatty Infiltration
ICC	Intraclass Correlation Coefficient
MRI	Magnetic Resonance Imaging
NDI	Neck Disability Index
PASS	Patient Acceptable Symptom State
SCM	Sternocleidomastoid
SD	Standard Deviation
VAS	Visual Analog Scale
