# Supplementary material for: Equivalent Clinical Outcomes but Divergent Biological Adaptation After Anterior Cervical Discectomy and Fusion Versus Cervical Disc Arthroplasty: A Longitudinal MRI Cohort Study
Source: J Clin Med. 2026 Aug 6;15(15):6116. doi: 10.3390/jcm15156116 (PMC13467554; doi:10.3390/jcm15156116)
Supplement: Supplementary file 1 [file jcm-15-06116-s001.zip › Table_S1.pdf]

# Equivalent Clinical Outcomes but Divergent Biological Adaptation After Anterior Cervical Discectomy and Fusion versus Cervical Disc Arthroplasty: A Longitudinal MRI Cohort Study

## Supplementary Materials

Table S1. Sex-stratified subgroup analysis of postoperative regional muscle change within each surgical group (exploratory; n = 25 per sex per group).

| Group | Muscle / parameter | Female | Male | p value | q (BH-FDR) |
|---|---|---|---|---|---|
| ACDF | Multifidus Δ CSA | -18.6% | -17.5% | 0.977 | 0.977 |
| ACDF | Multifidus Δ FI (pp) | +10.0 | +6.3 | 0.021 | 0.084 |
| ACDF | Semispinalis cervicis Δ CSA | -24.2% | -13.8% | 0.0009 | 0.007 |
| ACDF | Semispinalis cervicis Δ FI (pp) | +6.8 | +5.9 | 0.698 | 0.797 |
| ACDF | Semispinalis capitis Δ CSA | -19.7% | -10.3% | 0.0003 | 0.002 |
| ACDF | Semispinalis capitis Δ FI (pp) | +5.2 | +6.2 | 0.497 | 0.663 |
| ACDF | Splenius capitis Δ CSA | -17.5% | -13.7% | 0.138 | 0.275 |
| ACDF | Splenius capitis Δ FI (pp) | +5.9 | +4.3 | 0.067 | 0.178 |
| CDA | Multifidus Δ CSA | -9.1% | -9.0% | 0.545 | 0.749 |
| CDA | Multifidus Δ FI (pp) | +2.5 | +2.1 | 0.749 | 0.749 |
| CDA | Semispinalis cervicis Δ CSA | -6.8% | -7.4% | 0.666 | 0.749 |
| CDA | Semispinalis cervicis Δ FI (pp) | +1.9 | +0.9 | 0.028 | 0.222 |
| CDA | Semispinalis capitis Δ CSA | -4.8% | -5.7% | 0.259 | 0.749 |
| CDA | Semispinalis capitis Δ FI (pp) | +2.0 | +1.6 | 0.443 | 0.749 |
| CDA | Splenius capitis Δ CSA | -6.6% | -4.8% | 0.534 | 0.749 |
| CDA | Splenius capitis Δ FI (pp) | +1.1 | +1.1 | 0.573 | 0.749 |

CSA = cross-sectional area; FI = fatty infiltration; pp = percentage points. Δ CSA is the mean within-group percentage change from baseline to 12 months; Δ FI is the mean absolute change in percentage points. Comparisons are female versus male within each surgical group by Mann–Whitney U test; this subgroup analysis was designated exploratory a priori. Raw p values are shown alongside Benjamini–Hochberg false discovery rate-corrected q values computed across the eight comparisons within each group. In the ACDF group, sex differences remained significant after correction for semispinalis cervicis and semispinalis capitis Δ CSA (q = 0.007 and q = 0.002). In the CDA group, no comparison remained significant after correction (all q  0.22); the nominally significant raw p value for semispinalis cervicis Δ FI (p = 0.028) did not survive FDR correction.
